# Single visit versus multiple visit root canal

**DOI:** 10.6026/973206300161100

**Published:** 2020-12-31

**Authors:** Fahmida Binti Abd Rahman, Mahesh Ramakrishnan, MS Niveditha

**Affiliations:** 1 Saveetha Dental College, Saveetha Institute of Medical and Technical Sciences, Saveetha University, Chennai 77, India

**Keywords:** Canals, pulp necrosis, multi visits, single visit

## Abstract

Single visit endodontics offers many advantages over multi visit treatment. Therefore, it is of interest to assess the preference of single visit over multiple visit root canals. We used 86,000 patient records and selected 9017 records matching the inclusion criteria for the analysis using statistical tools (Chi square test at p value <0.05). Data shows that people between 26 to 45 years are often affected with dental caries. Available data is biased towards multi visits rather than single visit regardless number of canals.

## Background

Successful and proper root canal treatment influenced by adequate debridement and filling of the entire root canal system is important [1]. Factors such as missing additional root canal, improper cleaning and shaping cause the failure of the entire treatment, altogether bringing frustration to both clinician and patients. Hence, it is important to have great knowledge and be familiar with the variations in the root canal morphology [2]. Historically, multi visit root canal treatment was the only option for root canal treatment. The main objective for the multi visit was mainly to ensure sterility of the root canal system prior to obturation. As biomechanical preparation and irrigation were not possible to ensure complete sterilization, therefore application of intra canal medicaments were needed to eradicate the bacteria inside the canal system [3]. However, excess application of these medicaments could cause postoperative complications and persistent periradicular infections. As for this reason, clinicians had incorporated single visits also in their clinical practice especially for root canal treatment. However, not all cases should be done with a single visit. The selection whether to perform root canal treatment either in single visit or multiple visit mainly depends on case selection, presence or absence periradicular lesions, patients cooperation, clinicians skill and many more [5]. Single visit root canal offers many advantages such as it can decrease the number of operative procedures including additional anesthesia, gingival trauma from rubber dam application as well as eliminating the risk of inter appointment leakage through temporary restoration. Therefore, it is of interest to assess the preference of single visit over multiple visit root canal.

## Methodology

### Study design:

The present study was a retrospective study done in a university setting at the Department of Pediatric in a private dental college. A total of 86,000 case records of patients were evaluated and it was found that 9017 patients were selected and were included in the present study. The advantages for this study setting are it can provide easy accessibility to data and provide a population with similar ethnicity. The inclusion criteria would be all patients with history of undergoing root canal treatment meanwhile the exclusion criteria would be all the incomplete case sheets.

### Ethicals:

Before scheduling of the retrospective study, the official permission was obtained from the Institutional ethical committee (ethical approval number- SDC/ SIHEC/ 2020/ DIASDATA/ 0619-0320).

### Data collection:

Data selected was between the time periods of June 2019 to March 2020. The age groups were split into three categories which were <25 years old, 26-45 years old and >46 years old. The convenient sampling method was used and photographic verification was done for cross verification of data. Sample data were cross-verified by another examiner to avoid any missing data. Data regarding age, gender, number of canals, number of visits, type of teeth affected were retrieved from patient's records and tabulated in Microsoft Excel.

### Statistical analysis:

Data was analysed using SPSS software (IBM SPSS Statistics, Version 24.0, Amonk, NY: IBM Corp). Descriptive statistics were used for data summarization. Chi square test was done to test the relationship between the variables. The level for a statistical significance was set at a p value<0.05. Association between the number of canals and number of visits was analysed. The results were demonstrated in the form of bar graphs.

## Results and Discussion:

In the present study, a total of 86,000 case records of patients were evaluated and it was found that among them 9017 of them had a history of undergoing either single visit and multiple visit root canal treatment. In the patients treated with root canal treatment 4849(53.78%) of them were males and 4168(46.22%) were females (Figure 1 - see PDF). This finding indicates that male had slightly higher prevalence for dental caries in comparison to females. The variation in terms of findings between current study and other studies could be due to many factors such as study setting, ethnicity, socioeconomic status, diet, oral hygiene, sample size for both male and females in each study, time period of the study and many more [6- 8]. Based on the distribution of age of patients that underwent root canal treatment, it can be noted that patients aged 26-45years (47.72%) had higher prevalence for root canal therapy followed by patients aged <25 years old (31.71%) and finally the lowest prevalence for root canal was found to be patients age >65 years old (Figure 2 - see PDF). It can be observed from the results obtained that the prevalence of caries was highest in middle age populations. One of the reasons for the higher prevalence of dental caries in middle age populations could be due to the fact that they are busy with their work and they were not able to spend time attending dental clinics for general dental check up or for basic treatment like oral prophylaxis which was recommended twice per year [7-10]. Other reasons could be due to the lack of knowledge regarding signs and progression of dental caries, lack of dental care and many more. According to the distribution of the number of treatment visits, it can be noted that most of the endodontists preferred to perform root canal treatment in multiple visits (82.90%) in comparison to single visits 17.10% (Figure 3 - see PDF). One of the many advantages of completing root canal treatment in a single appointment would be there was little evidence regarding risk of flare up induced by leakage of the temporary seal between appointments and materials needed for separate visits could be saved up. According to the association between the number of canals and the number of root canal visits, it could be observed that multi visit was highly preferable among clinicians for root canal treatment in most of the cases regardless of the number of canals among the teeth. Chi square test was found to be statistically significant as the p value was 0.000, which was less than 0.05. The reason for this variation could be attributed to the fact that amount or bacteria can be decreased by application of additional use of calcium hydroxide as intra canal medicaments in multiple visit appointments [11]. Calcium hydroxide has been considered as one of the most important antimicrobial dressing and it was practically used by many clinicians in dental practise. Calcium hydroxide will provide an alkaline environment, which was not suitable for most of the endodontics pathogens. The antimicrobial activity of these medicaments could be explained by the release of hydroxyl ions in an aqueous environment [12-15].

## Conclusion

We show that people between 26 to 45 years are often affected with dental caries. Available data is biased towards multi visits rather than single visit regardless number of canals. More data will add value to this observation.
